# 

**Published:** 1875-12

**Authors:** 


### INDEX

### SOUTHERN MEDICAL RECORD, Volume V., January to December, 1875

PREPARED BY C. H. TIDD, M. D., GENEVA, IOWA.

Abdominal walls, passage of a scissors through the, etc., 367
Ablation of the breast by the elastic ligature, 371
Abortion, Dr. Paul F. Mundi's treatment of, 294
Abortion, removing the ovum in cases of, 619
Abscess, application of oleate of mercury in threatened mammary, 54
Abscess, mammary, 109, 252
Abscess of the penis, report of four cases, by E. T. Easley, A. M. M. D., 270
Abscess of liver, 538
Abscess of breast treated by compressed sponge, 624
Abscesses, on the prevention of mammary, etc., 478
Abscesses and boils, sulphide of calcium in scrofulous, by T. Curtis Smith, M. D., 409
A case of sudden death, 727
Academy of Medicine, action of, 253
Accumulation and inspissation of cerumen, by Swan M. Burnett, M. D., 587
Acacia, syrup of, 697
Acetic acid, in mucous polypus, 36, 117
Acid, muratic, 752
Acid, iodic, in hypodermic injections, 45
Acid, carbolic, in tapeworm, 53
Acid, carbolic, subcutaneous injections of, in traumatic erysipelas, 112
Acid, carbolic, treatment of pneumonia and bronchitis by, 119
Acid, carbolic, in cheesy pneumonia, the use of, 243
Acid, carbolic, arrest of erysipelas by means of injections of, 313
Acid, carbolic, and the hyposulphites, in scarlatina, 370
Acid, uric, in the organism, generators of, 663
Acid stains, removal of nitric, 118
Acid, benzoic, treatment of ammoniacal cystitis by, 241
Acid, salicylic, for ulcers, 309
Acid, salicylic, as a disinfectant, 427
Acid, salicylic, in extensive burns, 440
Acid, salicylic, solubility of, 441
Acid, salicylic, solubility of, in glycerin, 697
Acid, salicylic, 442, 501
Acid, salicylic, in diphtheria, 495
Acid tannate of iron in diphtheria, 693
Acidulated gargles in typhoid fever, 181, 488
Aconite in headache, 119, 166
Aconite, pills of, 314
Action of phosphorus as a stimulant, by John Brunton, M. A. M. D., 220
Actual cautery, 746
Acute rheumatism, 222
Acute articular rheumatism, 245
Acute hematuria treated successfully by local applications, 245, 496
Acute articular rheumatism, by C. H. Tidd, M. D., 449
Addison's disease, cause of, 740
Adhesion of the placenta, by J. G. Swayne, M. D., 602
Adhesive plaster, a new, 691
Administration of phosphorus, the, 48
Administration of purgatives by the hypodermic method, 494
Advantages and disadvantages of Esmarch's bloodless method, 157
Advertising department, our, 121
Advertising, medical, 572
Africa, a fly of, 184
Agent, clay as a therapeutic, by A. L. Knight, M. D., 14
Ailanthus glandulosa in dysentery, 182
Air, breathing in rarefied, 110
Air baths, treatment of typhoid fever by, 251
Albuminuric eclampsia, a case of, by G. L. Ullman, M. D., 397
Alkalinity of urine, hematoxylon in, 744
Allopathy and homœopathy, 571
Aloes and ipecac, pills of, 297
Aloeolaris, how to cure pyorrhœa, 626
Amalgamated placenta, a case of, 161
Amber, oil of, in hiccough and spasmodic affections, 45

Amenorrhœa, electricity in, 95
Amenorrhœa, pulsatilla in, 428
American surgery, Mr. Erichsen on, 178
American medical association, 189
Ammonia, muriate of, for nervous diseases, 34
Ammonia, bromide of, in catamenial excess, 432
Ammonium, bromide of, in catamenial excess, 680
Amyl, nitrite of, and belladonna in dysmenorrhœa, 167
Amyl, nitrite of, 304
Anæsthetics, influence of, upon the sexual impressions of females, 107, 683
Anæsthetic, bromide of potassium, as a caustic, etc., in tumors, 311
Anæmia, progressive pernicious, 289
A new method of administering chloral, *enemata*, 743
Ani, prolapsus of, treated by ergotin, 307, 363
Aniline in chorea, 742
Annointing with cocoa butter in scarlet fever, 52
Ano, fissures in, treated with iodoform, 168
Anodyne, a convenient local, 302, 444
Anomalous digestion, 426
Anthelmintic, action of kameela, 743
Anti-periodic, tincture nucis vomica as an, by W. W. Fierce, M. D., 7
Antidote to strychnine, nicotine as an, 41
Anti-septic ointments, new, 52, 314
Antidote for phosphorus, turpentine an, 171
Antagonism between chloral hydrate and calabar bean, 175
Antidote to strychnia, 195
Anti-gastralgic pills, 313
Anti-emetic properties of tobacco, 416
Antidote to chloroform, 424
Anti-gastralgic drops, 434
Anti-emetic, strychnia as an, 443
Anti-pruritic remedy, a new, by L. Duncan Bulkly, A. M. M. D., 598
Anti-catarrhal pills, 621
Anti-septic dressing, a new, 491
Anti-galactagogue, ergot as an, 493, 672
Anti-spasmodic pills, 748
Apo-morphine, 371
Applications of the forceps, 28
Aromatic syrup of senna, 246, 371
Arnica in orchitis, 734
Arsenic in malignant lympho-sarconia, 116
Arsenic, poisoning case of chronic, 165
Arsenic, the capacity of plants to absorb from the earth, 234
Arsenic in asthma, 251
Arsenic in large doses, treatment of chorea by, 440
Artichoke in rheumatism, 252
Artificial respiration, an easily effectual method—by J. B. Mattison, M. D., 529
Arteries, surgery of, 487
Ascorides, injections of cod-liver oil for, 113, 698
A simple method of removing teeth of children, 752
Aspirator in retention of urine, 372
Association, British medical, 498
Asthmatic mixture, 744
Asthma, treatment of spasmodic, 118
Asthma, treatment of acute and chronic bronchitis, and, 226, 498
Asthma, chloral subcutaneously in, 241
Asthma, arsenic in, 251
Asthma, treatment of spasmodic, 252
Asthma, grindelia robusta in, 363
Asthma, belladonna in spasmodic, 364
Asthma, 698
Atropia in phthisical sweating—by A. G. Smythe, M. D., 21
Atropia in ophtthalmic affections, 99
Atropia, treatment of salivation by, 119
Atropia for subcutaneous administration, morphine with, 374
Atropine, hypodermic injections of morphia and, 53, 126
Atrophy, diplopia and ptosis with syplitic paralysis and muscular, 107
Aural disease, death from eucephalic inflammation consequent upon—by Swan M. Burnett, M. D., 201
Auscultation, a difficulty in fœtal,
Ayer's hernia truss, 255, 383
Babies' sore eyes, by Henry W. Williams, A. M. M. D., 203
Baths, tréatment of typhoid fever by air, 251
Baths in syphilis, mercurial, 685
Baths, hot, 688
Belladonna and nitrite of amyl in dysmenorrhœa, 167
Belladonna in the treatment of profuse perspiration, 309
Belladonna in spasmotic asthma, 364
Beverage, the remedial use of sea-water as a, 436
Bicarbonate of soda in treatment of toothache, 420
Biliary calculi within the gall bladder, solubility of, by Ralph S. Goodwin, M. D., 667
Binder in parturient women, on the uses of the, by P. H. Clarke, M. D., 51
Bismuth in skin diseases, 4223

Bismuth and creosote in infantile vomiting, 740
Bladder, catarrh and paralysis of the, 243
Bladder, puncture of, for retension of urine, 428
Bloodless operations, 238
Bloodless method, five cases of operation by the, by Lewis Balch, M. D., 391
Blood, action of, mercury on the, 298
Boars, trichinæ in wild, 313
Board of health, the State, 381
Bone dust as food, 694
Boils, new treatment of, 236
Boils, sulphide of calcium in scrofulous abscesses and, by T. Curtis Smith, M. D., 409
Boldo, 244
Boston medical library, 502
Brain, concussion of the, 401
Brain, oxygen in congestion of, 750
Breathing, in rarified air, 110
Breast, ablation of elastic ligature, 371
Breast, abcess of, treated by compressed sponge, 624
Bright's disease, acute, 111
Bright's disease, a cure for, 180
British medical association, 498
Bromide of potassium, by W. F. Barr, M. D., 11
Bromide of potassium, in obstinate vomiting enemata of, 119, 492
Bromide of potassium in malarial fever, 302
Bromide potassium as a caustic, etc., in malignant suppurating tumors, 311
Bromide of potassium in obstinate vomiting, 440
Bromide of potassium in strychnine poisoning, 483
Bromide of potassium in enema, 735
Bromide of calcium, 100
Bromide of calcium in the nocturnal pains of syphilis, 314
Bromide of camphor, 167
Bromide of ammonia in catamenial excesses, 432, 680
Bromide of iron in chorea, failure of, 616,
Bromide of potassium and chloral in chest affections, 748.
Bromine in pertussis, 178
Bromide, local applications of, in treatment of carcinoma of cervix, 237
Bronchial catarrh, tar in, 725
Bronchitis, muriate of ammonia in, etc., 114
Bronchitis and pneumonia treated by carbolic acid, 119
Bronchitis and asthma, acute and chronic, treatment of, 226, 498
Broncho-pneumonia, a chemical lecture on typhoid fever and, by Francis Delafield, M. D., 340
Bronchial catarrh and winter croup, the value of tar in, 625
Brow-beating hysteria, 492
Bubo, suppurating, 231
Bubo, indurated, 252
Bullet wound of the stomach and kidneys, 503
Burns, liquid glycerine for, 315
Burns and scalds, 424
Burns, salicylic acid in extension, 440
Burnett, Dr. Swan M., 382
Byrd on cholera infantum, 101
Cæsarean operation successful for both mother and child, 179
Cæsarean section, in a case of uterine fibroids, successful, 369
Cæsarean operation, in ovariotomy complicated with pregnancy, 614
Calcium, bromide of, 101
Calabar bean and hydrate of chloral, antagonism between, 175
Calcium, bromide of, in nocturnal pains of syphilis, 314
Calcium, sulphide of, in scrofulous abscesses and boils, by T. Curtis Smith, M. D., 409
Calculi, solubility of biliary, in the gall bladder, by Ralph S. Goodwin, M. D., 667
Camphor water, 110
Camphor in erysipelas, 118,494
Camphor, bromide of, 167
Cancer, silica in, 160
Cancer, local treatment of, 690
Cantharides, the danger of, 696
Capitas, eczema, 50
Carbolic acid in tapeworm, 53
Carbolic acid, traumatic tetanus, subcutaneous injection of, 112
Carbolic acid, treatment of pneumonia and bronchitis by, 119
Carbolic acid, internally in proriasis, 230
Carbolic acid, in cheesy pneumonia, 243
Carbolic acid, arrest of erysipelas by injections of, 313
Carbolic acid and hyposulphites, in treatment of scarlatina, 370
Cardiac disease, 45
Cardiac œdema, 116, 183
Carcinoma of the cervix, local application of bromine in, 237
Carminative mixture for young infants, 306
Carbon, sulphuret of, in chronic and atonic ulcers, 438
Carbuncle, treatment of, 742
Carcinoma, 618

Cash payments to the Record, list of, 64, 128, 192, 255, 320, 383, 448, 639
Castor oil, administration of, 312
Catheterism, position for, 47
Catarrh, 56
Catarrhal jaundice, treatment of, by electricity, 172
Catarrh, nasal, 175
Catarrh and paralysis of the bladder, 243
Catarrh, intestinal, diet of infants in, 566
Catarrhal pills, anti, 621
Catarrhal affections, 696
Catarrh and winter cough, value of tar in bronchial, 625
Catheter impacted in female pelvis, 184
Catamenial excesses, bromide of ammonia in, 432
Cataract, requiring extraction, improved method of treating certain cases of, 559
Cataract, extraction of, by a median section through the cornea, 559
Causation of typhoid or enteric fever, 49
Cautery, galvano, 105
Caustic, bromide of potassium as a, etc., 311
Caustics, cure of vesico vaginal fistula by, 481
Cavities, the injection of pulmonary, 25
Cement, a good, 116
Cement, improved glycerin, 176
Cerebro-spinal meningitis, by Wm. Read, M. D., 276
Cerumen, accumulation and inspissation of, by Swan M. Burnett, M. D., 587
Certain sign of death, 503
Champion chloroform tippler, 111
Chancres, soft, 173
Charity vs. policy, 317
Charcot, on relief of hysterical seizures by compression of the ovaries, 494
Chest, tepid baths in diseases of the, 152
Chest affections, influence of use of wind instruments on, 565
Children, marasmus of, 6
Children, points in the treatment of diseases of, 35
Children, formula for diarrhœa and dysentery in, 54
Children, convulsions in, by Wm. T. Beall, M. D., 85
Children, teeth, simple method of removing, 370
Childbed, the mortality of, 549
Chills, chloroform in congestive, 300
Chills viewed in a new light, 741
Chill misplaced, 86
Chloral in gun-shot wounds, tetanus, etc., by A. R. Kilpatrick, M. D., 18
Chloral, hydrate as a solvent, 38
Chloral, hydrate in puerperal mania, 46
Chloral and ipecacuanna in croup, 56
Chloral drinking, delirium tremens after, 57
Chloral hydrate, chronic poisoning with, 115
Chloral hydrate to arrest putrefaction and fermentation, 174.
Chloral hydrate and calabor bean, antagonism between, 175
Chloral subcutaneously in asthma, 241
Chloral hydrate in hyper-emesis, 353
Chloral and morphia in tetanus, 373
Chloral suppositories, 426
Chloral, tetanus treated by, 622
Chloral, treatment of ozœna by injections of, 697
Chloral, 739, 741, 743
Chloral, a new method of enema, 743
Chloral and bromide of potassium in enemata, 738
Chloral and bromide of potassium in chest affections, 748
Chloroform in strychnia poisoning; 50
Chloroform as a liniment, 100
Chloroform narcosis, Nelaton's method of resuscitation from, 105
Chloroform tippler, the champion, 111
Chloroform, preservation of vegetable infusion by, 249
Chloroform in congestive chills, 300
Chloroform, an antidote to, 424
Chloroform in lead colic, 621
Chloroform, effect of the inhalation of. on the fœtus, during parturition, 629
Chloroform vs. ether, 695
Chloroform, history of, 748
Chlorate of potassa, fatal poisoning from, 55
Chlorate of potassa in ozœna, 241
Chloride of zinc, 368
Chlor-hydrate of morphia, the curative treatment of insanity by, 482
Chocolate in intestinal catarrh, 439
Cholera, 57, 117
Cholera infantum alias misplaced chill, 86
Cholera infantum, Byrd on, 101
Cholera, history of, summed up, 109
Cholera, remedy for, 306
Cholera, prescription for, 501
Chorea treated by arsenic in large doses, 440
Chorea, treatment of, as adopted by Anstie, 443
Chorea, failure of bromide of iron in, 616
Chorea a sequence of rheumatism? Is, by J. C. Bishop, M. D., 653

Chronic chorea, aniline in, 742
Cider, how to keep it sweet, 112
Cincho quinine, 300, 631, 633
Cinquefoil in puerperal peritonitis, 360
Circulation? What are the causes of the venous, by J. G. Knox, A. M. M. D., 582
Chronic hoarseness, remedy for, 46
Chronic ulcers, 244
Chronic ulceration of the middle ear, iodoform in, 437
Chronic rheumatism, guarana in, 438
Chronic intestinal catarrh, chocolate in, 439
Clay as a therapeutic agent, by A. L. Knight, M. D., 14
Claims of the medical profession, 32
Clinical notes, by F. K. Bailey, M. D., 394, 532
Cloasma uterina, tincture of iodine for, 355
Clysters, in dysentery iced. 235
Cocoa butter, in scarlet fever, anointing with, 52
Cod-liver oil for ascarides, injections of, 113, 698
Cod-liver oil jelly, 117
Cod-liver oil and phosphorus combined, 746
Cohosh in rheumatism, black, 46
Colic, lead, chloroform in, 749
Cold powder, 233
Colds, common, 684
Colleges, medical, 445, 508, 510
Colic, chloroform in lead, 621
Colic hepatic, cured by chloroform, by Q. C. Smith, M. D., 665
Collodion in the treatment of erythema and interrigo, 628
Compound syrup of garlic, 48
Communications received, 61, 126, 380, 382, 511
Confinement, keeping the bed after, 39
Constipation, gelatin suppositories in obstinate, 55
Constipation of females, pills for, 75
Convulsions in children, by Wm. T. Beall, M. D., 85
Convulsions, puerperal, treated by hypodermic injections of morphia, by John D. McGill. M. D., 265
Congestive chills, chloroform in, 300.
Contagion in disease, studies and experiments on the prevention of, 366
Concussion of the brain, 401
Contribution to the treatment of epistaxis, by Beverly Robinson, M D., 402
Contagion of puerperal fever, 406
Conjunctiva, iodoform as a remedy in affections of the cornea and, 469
Contagion, remarkable instance of scarlatinal, 495
Copaiba in dropsy, 429, 179, 312.
Corpuscle, is the white blood and the puas corpuscle identical, by T. Curtis Smith, M. D., 198
Cornea, extraction of cataract by a medin section through the, 559
Cornea and conjunction, iodoform as a remedy in diseases of the, 469
Cosmetic, harmless, 751
Cough, whooping, 55, 242, 309
Cough, to check vomiting from, 56
Cough of chronic phthisis, 298, 312
Cough and sweating in phthisis, 374, 500
Cough, whooping, treatment of, 630
Couchard, the, 305
Coxalgia successfully treated by the use of Sayer's hip joint splint, by J. A. Draughan, M. D., 486
Cramp of telegraph operators, 418
Crayons of iodoform, 747
Cremation, 110
Creosote and bismuth in infantile vomiting, 740
Croup, chloral and ipecacuanha in, 56
Croup, true membranous, by W. H. Vail M. D., 659
Croupous phneumonia, ergotin in, 239
Croton chloral, 108
Croton chloral hydrate in megrin, by Sidney Binger, M. D., 213
Croton chloral hydrate, 679
Cyanides in acute articular rheumatism, the, 437
Cyprepedeum or ladies' slipper as an emmenagogue, 611
Cystitis ammoniacal treated by burzois acid, 241
Cystic paralysis ergot, in, 434
Dauriana, 373, 481
Death from use of perchloride of iron, 57
Deaf ear, unstopping a, 118
Deafness cured by extraction of teeth, 176
Death from eucephalic inflammation consequent upon exural disease, by Swan M. Burnett, M. D., 201
Deaths from London fog, 264
Dead fœtus retained in uterus over four months, 338
Death after delivery without any lesion to account for it, 366
Deaths, 511
Delirium in typhoid fever, 49, 498
Delirium tremens after chloral drinking, 57
Delirium tremens, treatment of, 171, 435.

Delayed issue, 375
Delivery, perineal tumor of fœtus an impediment to, 615
Diaphoretic, new, 752
Diarrhœa of tpyhoid fever, 31
Diarrhœa and dysentery in children, formulas for, 54
Diarrhœa of phthisis, 246
Diarrhœa mixture, 249, 425
Diarrhœa and dysentery, salacin in, by T. Curtis Smith, M. D., 328
Diarrhœa of typhoid fever, treatment of, 356
Diarrhœa, opium or salicin in, 490
Diarrhœa, oxide of zinc, infantile in, 620, 627
Diabetes, 119, 170, 562
Diagnosis of disease of the liver, 431
Diagnosis of ovarian disease, 698
Diabetes, glycerin in the treatment of, 566
Diachylon plaster hyperiodrosis cured by, 623
Diet, infant, 31
Diet in rheumatism, nitrogenous, 432
Diet of infants affected with acute intestinal catarrh, 566
Diet in rheumatism, non-nitrogenous, 629
Differential diagonosis between the red blood corpuscle of human and certain lower animals, 180
Differential symptoms of epidemic sore-throat and diphtheria, by A. L. Knight, M. D., 577
Digitalis, 50
Digestion, anomalous, 426
Digitalis in typhoid fever, 435
Dilalation of the os uteri, as a means of inducing premature labor, manual, by A. D. Sinclair, M. D., 207
Dilalation of the os uteri in obstinate vomiting in pregnancy, 441, 628
Diminution of the uterus after delivery, 686
Diphtheria, 40, 53, 361, 420, 436
Diphtheria, lemon juice in, 156
Diphtheria, ice in, 240
Diphtheria, iodide of potassium in, 243
Diphtheria, phoryngeal, prescriptions for cures of, 315
Diphtheria, on the treatment of, 360
Diptheria, local treatment of, 401
Diphtheria, salicylic acid in, 496
Diphtheria, of a vaccination pustule followed by general diphtheria, 561
Diphtheria, differential symptoms of epidemic sore throat and, by A. L. Knight, M. D., 577
Diphtheria and scarlet fever, treatment of, 623
Diphtheria, acid tannate of iron in, 693
Diplopsia and ptosis with syphilitic paralysis and muscular atrophy, 107
Diseases of children, points in the treatment of, 35
Dispersion of tumors by puncture, 164
Dislocation of humerus after five months, 373
Dislocation of the shoulder, two new differential signs in, by Prof. Frank H. Hamilton, M. D., 547, 412
Disinfectant, salicylic a id as an, 427
Displacements of the uterus, 540
Double vagina and uterus, case of, by A. E. Hoadly, M. D., 661
Double pistula in ano treatment of, 737
Doctorate, a proposed standard for the, 362
Dolens phlegmasia, 86
Dressing, a new anti-septic, 491
Dressing for ulcers, 621
Dressing, new material for fixed, 691
Dropsy of the amnion, by J. C. Bishop, M. D., 72
Dropsy, copaiba in, 179, 312, 429
Drum membrane, another method of perforating, by Joseph Sinrock, M. D., 415
Drunkenness, Russian cure for, 444, 739
Duodenitis, by A. L. Knight, M. D., 641
Dysmenorrhœa, a case of membranous, by Dr. Unschuld, 22
Dysmenorrhœa, nitrate of amyl and belladonna in, 167
Dysmenorrhœa, remedy for, 229
Dysmenorrhœa, 232, 308
Dysmenorrhœa, valerianate zinc in, 751
Dysentery cured without opium, 166
Dysentery, ailanthus glandulosa in, 182
Dysentery, iced clysters in, 235
Dysentery, 250
Dysentery, salicine in diarrhœa and, by T. Curtis Smith, M. D., 328
Dyspepsia of smokers, 567
Ear, extraction of foreign bodies from the, 170
Ear, eczema of the, 229
Early operation for hare-lip, 352
Easy parturition, 112
Eating, sciatica, cured by, 630
Eclompsia, a case of albuminuric, by G. L. Ullman, M. D., 397
Eczema capitis, 50
Eczema of the ear, 229
Eczema, treatment of, 472
Eczema, 743
Editorial and miscellaneous, 58, 120, 185, 253, 317, 375, 444, 504, 568
Elastic ligature, treatment of fistulous sinuses by the, 169
Elastic ligature, 231

Elastic ligature, ablation of the breast by the, 371
Elastic stocking, substitute for, 731
Electricity in amenorrhœa, 95
Electricity, treatment of catarrhal jaundice by, 172
Electricity, different degrees of susceptibility to, by Geo. W. Bread, M. D., 344
Emesis, hydrate chloral in hyper, 353
Emmenagogue, cyprepedeum or ladies' slipper as an, 611
Eucephalic inflammation, etc., death from, by Swan M. Burnett, M. D., 201
Encouragement, 574
Enemata of bromide of potassium in obstinate vomiting, 119, 492
England, longevity in, 501
Enteric fever, causation of typhoid or, 49
Epis taxis, treatment of, 175, 402
Epilepsy cured by excision of a neuroma, 177
Epithelioma, wood sorrel in, 439
Epidemic sore throat and diphtheria, differential symptoms of, by A. L. Knight, M. D., 577
Ergot in post-partum hemorrhage, hypodermic injection of, 183
Ergot internally in uterine polypi, by Daniel F. Collins, M. D., 286
Ergot in acute mania, 313
Ergot in cystic paralysis, 434
Ergot as an anti-galactagogue, 493, 672
Ergot, by T. Curtis Smith, M. D., 646
Ergot, modes by which it destroys uterine fibroids, 689
Ergotin in croupous pneumonia, 239
Ergotin, prolapsus ani treated by, 307. 363
Ergotin in uterine fibroids, 444
Erichsen on American surgery, Mr., 178
Error corrected, 637
Erysipelas, 44, 56
Erysipelas, camphor in, 118, 494
Erysipelas, treatment of, 223, 475
Erysipelas arrested by means of injections of carbolic acid, 313
Erysipelas, external use of tincture of iron in, 425
Erysipelas, cellulo cutaneous, 719
Erysipelas, pathological anatomy of, 723
Erythema and intertrigo treated by means of collodion, 628
Esmarch's operation, 299
Esmarch's method, critique on the, 489
Ether for tape worms, 316
Ether vs. chloroform, 695
Ether, threatened death from, 736
Ethiops mineral, 630
Eucalyptus and tobacco, 493
Excision of upper extremity of femur, after compound coruinanuated fracture, by P. H. Harris, M. D., 257
Exchanges, notice to our, 60
External use of turpentine in the treatment of tonsilitis, 51
Extraction of teeth, deafness cured by the, 176
Eyes, the hygiene of, 296
Eyes and spectacles, old, by Swan M. Burnett, M. D., 385
Eye, extraction of foreign bodies from the, 365
Facial neuralgia, gelseminum in odontalgia and, 695
Failure of bromide of iron in chorea, 616
Fatal poisoning from chlorate of potassa, 55
Fatal obstetrical cases, two, by John Hockenhall, M. D., 79
Female, hydrocele in a, by G. A. Baxter, M D., 83
Femur, excision of the upper extremity of, after compound coruinanuated fracture of the, by P. H. Harris, M. D., 257
Ferruginous preparation, elegant, 752
Fever, the diarrhœa of typhoid, 31, 356
Fever, typhoid, 43, 169
Fever? Why do we give wine in, 44
Fever, causation of typhoid or enteric, 49
Fever, delirium in typhoid, 49, 498
Fever, ulceration of vagina, larynx, etc., in typhoid, 51
Fever, anointing with cocoa butter in scarlet, 52
Fever, treatment of typhoid, 111
Fever, acidulated gargles in typhoid, 181, 488
Fever, treatment of typhoid by air-baths, 251
Fever, new method of treating typhoid, 293
Fever, bromide of potassium in malarial, 302
Fever, a clinical lecture on broncho-pneumonia and typhoid, by Francis Delafield, M. D., 340
Fever, puerperal, is it contagious? 368
Fever, puerperal, the contagion of, 406
Fever, typhoid, digitalis in, 435
Fever, typhoid, inunction in, 737
Fever, puerperal, 555
Fever, treatment of diphtheria and scarlet, 623
Fever, intermittent, sulphate cinchonidia in, 732
Fibroids, successful Cæsarean section in a case of uterine, 369
Fissures in ano treated with iodoform, 168

Fistulous sinuses treated by the elastic ligature, 169
Fistula, a new instrument for operating on vesico-vaginal, 558
Flooding, Indian hemp in, 564
Fly of Africa, a, 184
Fœtus, retained in utero over four months, a dead, 338
Fœtus, life in a six months', 367
Fœtus, perineal tumor of, an impediment to delivery, 615
Fœtus, effect of the inhalation of chloroform, during parturition on the, 629
Food for infants, oatmeal farina as a, 51
Food, case of abstention from, 297
Food, infant, 310
Foot bath, in urticaria, mustard, 370
Forceps, application of the, 28
Formula for intertrigo, 47
Foreign bodies from the ear, extraction of, 170
Foreign body in a strictured osophagus, lodgement of a, 335
Foreign bodies from the eye, removal of, 365
Foreign bodies from the throat, removal of, by injections, 489
Fractured patella, treatment of, 433
Fractures, plaster Paris in the treatment of, by E. H. Trickle, M. D., 521
Fucus poultices 430
Galvano, cautery, 105
Galactorrhœa, treatment of, 312
Galactagogue, ergot as an anti, 493, 672
Gall bladder, solubility of biliary calculi within the, by Ralph S. Goodwin, M. D., 667
Garlic, compound syrup of, 48
Gargles in typhoid fever, acidulated, 181, 488
Gargle for sore throat, 695
Gastralgic pills, anti, 313
Gastralgic drops, anti, 434
Gelatin suppositories in obstinate constipation, use of, 55
Gelseminum in odontalgia and facial neuralgia, 695
Genitals, pruritus of the, 358
Generators of uric acid in the organism, 663
Georgia, Medical Association of, 185
Georgia Medical Society, action of the Middle, 190
Ghosts, syphilitic, 567
Gleet, prostatic, 299
Glue, to prevent, from becoming sour and mouldy, 29
Glycerite of morphia, chloral, 38
Glycerite of veratria, chloral, 38
Glycerite of morphia and camphor, chloral, 38
Glycerin cement, improved, 176
Glycerin for burns, lime, 177, 315
Glycerin sichel, 184
Glycerin in the treatmeut of diabetes, 566
Glycerin in medical pastes, 625
Glycerin, solubility of salicylic acid in, 697
Goa powder in skin diseases, 503
Gonorrhœa, local remedies in, 115
Gonorrhœa, 225, 252
Gonorrhœa, silicate of soda in, 238
Gonorrhœa, injections in, 314
Gonorrhœa, hydrastin in, 431
Gonorrhœa, treatment of, 627
Granular lids, iodoform in, 42
Greetings, glad, 59
Grindelia robusta, in asthma, 363
Growth and nutrition of the body, by Joshua B. Graves, M. D., 65
Guaiacum in the treatment of acute tonsilitis, 158
Guaiacum in acute diseases of the throat, 182
Guarana, in chronic rheumatism, 438
Gum cutting, by Charles E. Buckingham, M. D., 149
Gunshot wound, by A. R. Kilpatrick, M. D., 18
Gurjun oil in leprosy, 42
Hare-lip, early operation for, by E. R. Maxon, M. D., 352,
Hat, poisoned by a, 622
Hay fever, formula for, 316
Heat from the moon, 109
Headache, aconite in, 119, 166
Healing ulcers, a new method for, 561
Heart diseases, treatment of, 563
Heart disease, hygiene of, incurable, 563
Hematuria, acute, treated successfully by local applications, 245, 496
Hemorrhage, post-partum, by F. K. Bailey, M. D., 532
Hemorrhage post partum, treatment of, 72
Hemorrhage from the mouth and other mucous surfaces, by F. K. Bailey, M. D., 533
Hemorrhage, persistent, 242, 698
Hemorrhoids treated by injections of ergot, 248.
Hemp, Indian, in flooding, 564
Hernia, a case of strangulated, by J. W. Wimbish, M. D., 20
Hernia, a new mode of taxis for strangulated, by Augustus F. Erich, M.D., 154
Hernia, a mode of reducing strangulated, 246

Hernia truss, Ayer's, 255, 383
Hernia, the tractile method in,. 305
Hernia, a radical cure for, 476
Herpes Zaster, soothing application in, 227
Herpes Zaster, induced current for, 750
History of cholera summed up, 109
Hoarseness, remedy for chronic, 46
Horse-hair for sutures, 172, 624
Hospital board, a shameful demand by a, 127
Hot water injections in uterine disease, 26
ot water in surgery, warm and, by C. Henry Leonard, M. D., 607
Hot Baths, 688
Humerus, dislocation of, after five months, 373
Hydrate of chloral as a solvent, 39
Hydrate of chloral in puerperal mania, 46
Hydrate chloral, chronic poisoning by, 115
Hydrate of chloral to arrest putrefaction and formentation, 171
Hydrate of chloral and calabar bean, antagonism between, 175
Hydrate of chloral in hyper-emesis, 353
Hydro-chloral by the rectum in vomiting of pregnancy, 421
Hydrate-croton chloral, 679
Hydrarthrosis, treatment of, 232
Hydrocele in female, by G. A. Baxter, M. D., 83
Hydrocele of the neck, by Sampson Gangee, F. R. S. E., 417, 485
Hydrastin in gonorrhœa, 431
Hygiene of the eyes, 296
Hygiene of incurable heart disease, 563
Hypodermic medication, by J. B. Mattison, M. D., 1, 332
Hypodermic injections, iodic acid in, 45
Hypodermic injections of morphia and atropia, 53
Hypodermic injections of morphia, solution for, 54, 364, 697
Hypodermic injections of ergot in postpartum hemorrhage, 183
Hypodermic injections, by D. McLean Forman, M. D., 267
Hypodermic injections, solution for, 501
Hypodermic injections, 671
Hypodermic injections of pure water, 751
Hypodermic use of morphia, puerperal convulsions treated by, by John D. McGill, M. D., 265
Hypodermic medication, 374
Hypodermic method, administration of purgatives by the, 494
Hyper-emesis, hydrate of chloral in, 353
Hyposulphites and carbolic acid in scarlatina, 370
Hyperidrosis cured by local application of diachylon plaster, 626, 749
Hysterical spasms, 244
Hysterical seizures treated by compression of the ovaries, 494
Hysterical seizures, 747
Hysteria, brow beating, 492
Iced clysters in dysentery, 235
Ice in diphtheria, 240
Idopathic tetanus treated by chloral, 304
Iodoform in granular lids, 42
Iodoform, fissures in ano treated with, 16e
Iodoform in chronic suppuration of th8 middle ear, 437
Iodoform in affections of the cornea and conjunctiva, 469
Iodic acid in hypodermic injections, 45
Iodide of potassium, a hint in giving, 311
Iodide of potassium, pills of, 312
Iodine, tincture of, for cloasma uterina, 355
Iodine as a substitute for iodide of potassium, 497
Ipecac and chloral in croup, 56
Ipecac and aloes, pills of, 297
Ipecac by inhalation, 769
Inanition, lacto phosphate of lime in mineral, 44
Incontinence of urine in paralysis, 38
Incontinence of urine, 443, 623, 554, 626
Incontinence of urine, subcutaneous injections of strychnine in, 692
Indian hemp in flooding, 564
Infant diet, 31
Infants, oat-meal farina as food for, 52
Infant food; 310
Infants, ointment for snuffles in, 565
Infants affected with acute intestinal catarrh, diet of, 566
Infantile diseases, quinine in the treatment of, 500
Infantile diarrhœa, oxide of zinc in 620
Inflammation of joints, persistent, 43
Influence of use of wind instruments on chest affections, 565
Ingrafting of tendons, 434
Ingrowing toe-nail, 692
Inhalations, treatment of pertussis by, 315, 365
Inhalation of chloroform during parturition, effect of, on the fœtus, 629
Injections of pulmonary cavities, 25
Injections of hot water in uterine disease, 26

Injections, iodic acid in hypodermic, 45
Injections of morphia aud atropia, hypodermic, 53
Injections of carbolic acid in traumatic tetanus, subcutaneous, 112
Injections of cod-liver oil for ascarides, 113
Injections of ergot in hemorrhoides, 248
Insanity, curative treatment by chlor-hydrate of morphia, 482
Insanity, ergot in the treatment of, 696
Insecticides, 499
Intertrigo, formula for, 47
Internal use of carbolic acid in proriasis, 230
Intestinal catarrh, use of chocolate in chronic, 439
Intestinal catarrh of infants, diet in the, 566
Intertrigo and erythema treated by means of collodion, 628
Iritis, by J. O. Stilson, M. D., 283
Iridotomy and its applications as a remedy for certain defects of the eye, by A. W. Calhoun, M. D., 321
Iron, death from the use of perchloride of, 57
Iron in erysipelas, external use of tincture of, 425
Iron in chorea, failure of bromide of, 616
Iron in diphtheria, acid tannate of, 693
Jaborandi, some of the therapeutic properties of, 354, 563
Jaundice, a pill for, 315
Jelly, cod-liver oil, 117
Juice of lemon in diphtheria, 156
Koumis, the use of, 106
Koumis, preparations of, 183
Labors, induction of premature, 105
Labor lingering from rigidity of the soft structures, 694
Lacto phosphate of lime in mineral inanition, 44
Lacerated wound of posterior perinarum, by E. T. Henry, M. D., 84
Ladies' slipper as an ammenagogue, cyprepedium in, 611
Larynx, ulceration of, etc., in typhoid fever, 51
Lead in soda water, 114
Leeches, the preservation of, 247
Lemon juice in diphtheria, 156
Lentil meal, 64
Leprosy, gurjun oil in, 42
Lichen, syphilitic, 237
Lichen, urticatus, local treatment of, 567
Lids, iodoform in granular, 42
Ligature, treatment of fistulous sinuses by the elastic, 169
Ligature, elastic, 231
Ligature, ablation of the breast by the elastic, 371
Lime glicerin for burns, 177
Liniment, chloroform as a, 100
Liniment for scabies, new, 174, 313, 630
Liniment, transparent turpentine, 307
Liniment, croton oil, 736
Liver, diseases of the, 97
Liver, diagnosis of diseases of the, 431
Liver, abscess of the, 538
Liver, traumatic inflammation of the, 693
Liquid, glycerin for burns, 315
Local treatment of cancers, 690
London fog, death from, 264
London doctors, 635
Lymphorrhagia, a case of, 564
Laxation of the penis, 682
Magendie's solution of morphia, 440
Malarial poisoning, 97
Malignant lympho sarcoma, arsenic in, 116
Malpractice, singular case of, 296
Malarial fever, bromide of potassium in, 302
Male girl, a, 496
Mammary abscess, threatened, 54
Mammary abscess, 109, 252
Mammary abscess, prevention of by application of the principles of rest, 478
Mania, puerperal, 46
Manual dilation of the os uteri to produce premature labor, by A. D. Sinclair, M. D., 207
Mania, ergot in acute, 313
Marsh mallow, new application of, 106
Mastitis treated with phytolacca decandra, by T. Curtis Smith, M. D., 536
Meal, lentil, 54
Measles and scarlatina, inunction as a remedy in, 307
Measures, standard weights and, 372
Medical profession, the claims of the, 32
Medicines, the stamp act on, 245
Medicines, explosive, 308
Medicines, preventive, 357
Medical organization, 462
Medical societies and medical journals, 568
Medical societies in Richmond, meetings of, 570
Medical advertising, 572
Medicines must not be shaken, homœopathic, 629
Medicated milk, 696
Megrim, chroton chloral hydrate in, by Sidney Ringer, M. D., 213
Membranous croup, treatment of true, by W. H. Vail, M. D., 659

Menorrhagia, 250
Meningitis, cerebro-spinal, by Wm. Read, M. D., 276
Menstruation, ovulation without, 491
Menses, bromide of ammonia in excessive, 680
Mercury, oleate of, and morphia in inflammations of the joints, 43
Mercury, action of, upon the blood, 298
Meterorism, production of, 734
Milk as a remedy, 98
Milk as a vehicle of disease, 310
Milk, prevention of the formation of, 495
Mineral inanition, lacto-phosphate of lime in, 44
Miracle, another, 628
Modern medical treatment, 240
Moon, heat from the, 109
Morphia and atropia, hypodermic injections of, 53
Morphia with atropia for subcutaneous administration, 374
Morphia, solution of, for hypodermic injections, 54, 364, 697
Morphia and quinine, 115
Morphia subcutaneously injected, action of, 251
Morphia, puerperal convulsions treated by the hypodermic use of, by John D. McGill, M. D., 265
Morphia and chloral in tetanus, 373
Morphia, Magendie's solution of, 440
Morphia, curative treatment of insanity by chlor-hydrate of, 482
Mortality of child-bed, 549
Mucous polypi, acetic acid in, 36, 117
Muriate of ammonia in nervous diseases, 34
Muriate of ammonia in bronchitis, 114
Muriatic acid, 752
Muscular atrophy, syphilitic paralysis and, 107
Mustard foot-baths in urticaria, 370
Musco volitentes, by John Coston, M. D., 656
Nævi, scarless eradication of, 676
Norcosis, Nelaton's method of resuscitation from chloroform, 105
Nasal catarrh, 175
Nasal polypi, tr. mur. ferri in, 295
Nausea of pregnancy, by F. K. Bailey, M. D., 394
Neck, hydrocele of the, 485
Nervous diseases, muriate of ammonia in, 34
Nervous diseases, nux-vomica in, 423
Neuralgia, for uterin , 47
Neuralgia, 104
Neuralgia, treatment of persistent, 176, 314
Neuralgia and rheumatism, 248
Neuralgia, phosphorus in, 487
Neuroma, epilepsy cured by excision of a, 177
Nicotine as an antidote to strychnia, 41
Night-mare, enlargement of the tonsils as a cause of, 57
Night-sweats of phthisis, 242
Nitrite of amyl, 304
Nitrogenous diet in rheumatism, 432
Nocturnal pains of syphilis, bromide of calcium in, 314
Non-nitrogenous diet in rheumatism, 629
Nucis vomicæ as an anti-periodic tincture, by W. W. Fierce, M. D., 7
Nutrition of the body, growth and, by Joshua B. Graves, M. D., 65
Nux-vomica in nervous diseases, 423
Obstetrical cases, two fatal, by John Hockenhull, M. D., 79
Obstetric swing, the, 107
Obstinate vomiting, enemata of bromide of potassium in, 119, 492
Oedema, cardiac, 116, 183
Odontalithus, by J. M. Lucas, D. D. S., 198
Odontalgia and facial neuralgia, gelseminum in, 695
Oil, cod-liver, injections of, for ascarides, 113
Oil, cod-liver, jelly, 117
Ointment, a new anti-septic, 314
Ointment, for sycosis, 435
Ointment for snuffles in infants, 565
Ointment for sycosis, 695
Old vs. the new, the, by "Old Fogy," 597
Onychia maligna, 17, 229
Ophthalmia, practical observations in, by J. O. Stilson. A. M. M. D., 279
Opium, dysentery cured without, 166
Opium, compound tincture of, 249
Opium, narcosis, external stimuli in, by J. B. Matison, M. D., 336
Opium or salicine in diarrhœa, 490
Orchitis, chronic, 240
Orchitis, arnica in, 734
Organization, medical, by A. A. Lyon, M. D., 462
Organism, generators of uric acid in the, 663
Os uteri, manual dilation of, etc., 207, 441, 628
Otto of roses, 239
Ovarian disease, pathology and treatment of, 87
Ovarian disease, diagnosis of, 698
Ovariotomy complicated with pregnancy, 614

Ovariotomy under difficulties, successful, 688
Ovalation without menstruation, 491
Ovum, removal of, in cases of abortion, 619
Oxide of zinc in infantile diarrhœa, 620, 627
Ozœna, chlorate of potash in, 241
Ozœna treated by injections of chloral, 697
Paralysis, incontinence of urine in, 38
Paralysis caused by worms, 100
Paralysis and catarrh of the bladder, 243
Paralysis, ergot in cystic, 434
Parturient, quinia as a, by W. B. Washington, M. D , 93
Parturition, easy, 112
Parturition, quinine in, 442
Parturient women, uses of the binder in, by P. H. Clarke, M. D., 513
Pathology of ovarian disease, 87
Patella, treatment of fractures of the, 433
Pathognomonic symptom of the moribund condition, 490
Pediculi, 27
Pelvis, catheter impacted in the female, 184
Penis, abscess of the, with four cases, by E. T. Easley, M. D., 270
Penis, laxation of the, 682
Perchloride of iron, death from, 57
Perspiration, belladonna in profuse, 309
Pertussis treated by inhalation, 315, 365
Personal, 619
Peritonitis, cinque foil in puerperal, 360
Peritonitis following retroversion of the uterus. 594
Percussion respiratory, 675
Perineurum, lacerated wound of, by E. T. Henry, M. D., 84
Perineal tumor of fœtus an impediment to delivery, 615
Persistent inflammation of joints, 43
Pernicious anæmia, progressive, 289
Phagedena, 248
Pharyngeal diphtheria, prescription of cures for, 315
Phlegmasia dolens, 86
Phosphorus, administration of, 48
Phosphorus, turpentine as an antidote for, 171
Phosphorus as a stimulant, by John Brunton, M. D., 220
Phosphorus in neuralgia, 487
Phosphorus as a stimulant, 497
Phosphide of zinc, 484
Phthisical sweating, atropia in, by A. G. Smythe, M. D., 21
Phthisis, treatment of, 41
Phthisis, night-sweats of, 242
Phthisis, diarrhœa of, 246
Phthisis, chronic cough of, prescription 298, 312
Phthisis, cough and sweating in, 374
Physician as a witness, 168
Physicians, satire on, the first authentic, 502
Phytolacca decandra, mastitis treate with, by T. Curtis Smith, M. D., 536
Pills of aloes and ipecac, 297
Pills of iodide of potassium, 312
Pills of aconite, 314
Pills. anti-gastralgic, 313
Pills for jaundice, 315
Pills of, sugar coated, 751
Pills of croton and chloral, hydrate, 746
Pills of, for constipation of females, 751
Pills of (grains), de sante, 747
Pityreasis versicolor, 174
Placenta previa, 79
Placenta amalgamated, 161
Placenta retained, 442, 698
Placenta, adhesion of the, by J. G. Swayne, M. D., 602
Plaster-paris in the treatment of fractures, by E. H. Trickle, M. D., 521
Plaster, a new adhesive, 691
Pleurisy, treatment of, 247
Pneumonia and bronchitis treated by carbolic acid, 119
Pneumonia, croupous, ergotin in, 239
Pneumonia, cheesy, carbolic acid in, 243
Pneumonia, typhoid fever and broncho, by Francis Delafield, M. D., 341
Podophyllin, new researches on, 113
Poisoning, case of chronic arsenic, 165
Poisoning, potassium bromide in strychnine, 483
Poisoning by strawberries, 566
Poisoning by opium of a suckling babe through the mother, 575
Poisoned by a hat, 622
Polk-root, tincture of, 369
Polypus of the uterus treated by ergot internally, by Daniel F. Collins, M. D., 286
Polypi, tr. mur. ferri in nasal, 295
Polypus, acetic acid in mucous, 36, 117
Post-partum hemorrhage, hypodermic injection of ergot in, 183
Post-partum hemorrhage, 532
Potassium, bromide of, by W. T. Barr, M. D., 11
Potassium, bromide, in malarial fever, 302
Potassium, bromide, as a caustic, etc., in suppurating tumors, 311

Potassium, bromide, in obstinate vomiting, 440
Potassium, bromide, in strychnia poisoning, 483
Potassium, bromide, in obstinate vomiting, enemata of, 492
Potassium, iodide of, in diphtheria, 243
Potassium, iodide of, a hint in giving, 311
Potassium, iodide of, pills of, 312
Potassium, iodide of, iodine as a substitute for, 497
Potassa, chlorate, fatal poisoning from, 55
Potassa, chlorate, in ozœna, 241
Powder, cold, 233
Premature labors, induction of, 105
Pregnancy, prolonged, etc., by A. R. Kilpatrick, M. D., 338
Pregnancy, nausea of, by F. K. Bailey, M. D., 394
Pregnancy, hydrochloral by the rectum in vomiting of, 421
Pregnancy, obstinate vomiting in, treated by dilation of the os-uteri, 441, 628
Pregnancy, facial discoloration in, 750
Pregnancy, puerperal, precaution against infection, 733
Premature baldness, 624
Prize, the Warren, 699
Prolapsed rectum, 52
Progressive, pernicious anæmia, 289
Prostalic gleet, 299
Prolapsus ani treated with ergotin, 307, 363
Pruritus vaginæ, 234
Pruritus, sulpho-carbolate of zinc in, 251
Psoriasis treated by the internal use of carbolic acid, 230
Ptosis with syphilitic paralysis, etc., 107
Puerperal convulsions treated by the hypodermic use of morphine, by John D. McGill, M. D., 265
Puerperal peritonitis, cinquefoil in, 360
Puerperal fever; Is it contagious? 368
Puerperal fever, contagion of, 406
Puerperal fever, 555
Pulmonary cavities, the injection of, 25
Pulmonary phthisis, tepid baths in, 152
Pulsatilla, amenorrhœa in, 428
Puncture of the bladder for retention of urine, 428
Purgatives by the hypodermic method, 494
Pyemia, 217
Pyorrhoea alvealaris, how to cure, 626
Pyrosis, 48
Quinia, as a parturient, by B. H. Washington, M. D., 93
Quinia and morphia, 115
Quinia in suppuration, by Douglas Morton, M. D., 146
Quinia in parturition. 442
Quinia, unchangeable solution of, for hypodermic injection, 315
Quinia as an oxytocic, 716
Rabies canina, by Alban S. Paine, M. D., 129
Radical cure for hernia, 476
Rattlesnake poison, by Alban S. Paine, M. D., 129
Raw meat, new mode of administering, 316, 443
Rectum, its relation to uterine diseases, the, by Arthur W. Edis, M. D., 163
Rectum, hydrochloral by, in the vomiting of pregnancy, 421
Relation of infant diet to gastro-intestinal diseases, by T. Curtis Smith, 705
Respect, tribute of, death of John G. Pepper, M. D.. 192
Respiration, easily effectual method of artificial, by J. B. Mattison, M. D., 529
Retention of urine, puncture of the bladder for, 428
Retained placenta, 442
Reviews of books received, 62, 127
Rheumatism, black cohosh in, 46
Rheumatism, acute, 222
Rheumatism, 235
Rheumatism, acute articular, 245
Rheumatism and neuralgia, 248
Rheumatism, artichoke in, 252
Rheumatism, nitrogenous diet in, 432
Rheumatism, non-nitrogenous diet in, 629
Rheumatism, acute articular, cyanides in, 437
Rheumatism, chronic, guarana in, 438
Rheumatism' acute articular, by C. H. Tidd, M. D., 449
Rheumatism, cerebral, treatment of, 739
Roses, otto of, 239
Russian cure for drunkenness, 444
Saccherated Dover powder, 696
Salicylic acid for ulcers, 309
Salicylic acid as a disinfectant, 427
Salicylic acid in extensive burns, 440
Salicylic acid, solubility of, 441
Salicylic acid, 442. 501
Salicylic acid in diphtheria, 496
Salicylic acid,solubility of, in glycerin,697
Salicine in diarrhœa and dysentery, by T. Curtis Smith, M. D., 328
Salicine or opium in diarrhœa, 490
Satire on physicians, the first authentic, 502

Sayers' hip-joint splint, coxalgia treated by the use of, 486
Scabies, a new liniment for, 174, 313
Scarlatina, the wet sheet in, 227
Scarlatina treated by hypo-sulphites and carbolic acid, 370
Scarlatina, sulpho-carbolate of sodium for, 742
Scalds, burns and, 424
Scarless eradication of nævi, 677
School for nurses, 630
Scissors, passage of a, through the abdominal walls after being swallowed, 367
Sciatica, 444
Sciatica, cured by eating, 630
Scriptures, the new, 123
Scrofulous abscesses and boils, sulphide of calcium in, by T. Curtis Smith, M. D., 409
Sea sickness, prevention of, 359
Sea water as a beverage, remedial use of, 436
Senna, aromatic syrup of, 246, 371
Sexual impressions of females, influence of anæsthetics upon the, 107
Shoulder, two new differential signs in dislocations of the, by Prof. Frank H. Hamilton, M. D., 412, 547
Sign of death, certain, 503
Silicated cotton, wounds dressed with, 30
Skin disease, bismuth in, 422
Skin disease, goa powder in, 503
Sleep, to promote tranquil, 113, 698
Small-pox, to prevent the marks of, 497
Smokers, the dyspepsia of, 567
Soda water, lead in, 114
Soda silicate in gonorrhœa, 238
Soft structures, lingering labor from rigidity of the, 694
Solvent, hydrate of chloral as a, 38
Sore throat and diphtheria, differential symptoms of, by A. L. Knight, M. D., 577
Spasmodic affections, oil of amber in, 4
Spasms, hysterical, 244
Spasmodic asthma, treatment of, 252
Spanish, characteristically, 441
Spermatorrhœa, treatment of, by a double truss, 159
Spectacles, old eyes and, by Swan M. Burnett, M. D., 385
Sponge tents, preparation of, 181
Sprains, 247
Stamp act on medicines, 245
Standard weights and measures, 372
State board of health, 381
Sterility, 503
Stimulant, phosphorous as a, by John Brunton, M. D., 220
Story, a strange, 173
Stomach and kidney, bullet wound of, 503
Strangulated hernia, a case of, by J. W. Wimbish, M. D., 20
Strangulated hernia, a mode of reducing, 246
Strychnine poisoning, chloroform in, 50
Strychnine as an anti-emetic, 443
Strychnine poisoning, bromide of potassium in, 483
Strychnine in incontinence of urine, subcutaneous injections of, 692
Strumarium xonthium, 500
Strawberries, poisoning by, 566
Strapping, wet, 613
Styptic collodion in erysipelas, 56
Subscribers, to our delinquent, 631, 699
Subcutaneous injections of carbolic acid in traumatic erysipelas, 112
Sulpho-carbolate of zinc in pruritus, 251
Sulphide of calcium in scrofulous abscesses and boils, by T. Curtis Smith, M. D., 409
Sulphuret of carbon in chronic and atonic ulcers, 438
Suppositories, chloral, 426
Suppuration of the middle ear, iodoform in chronic, 437
Suppression of urine, retention and, by R. J. Preston, M. D., 459
Surgery, progress of, 96, 487
Surgery, new principles of, 301
Surgery, warm and hot water in, by C. Henry Leonard, M. D., 607
Sutures, horse-hair for, 172, 624
Sweating, atropia in phthisical by A. G. Smythe, M. D., 21
Swing, the obstetric, 107
Sycosis, ointment for, 435, 695
Symptom of the moribund condition, pathognomonic, 490
Syphilis, the curability of, 106
Syphilis, bromide of calcium in the nocturnal pains of, 314
Syphilis, secondary, 689
Syphilis, congenital, in infants, 729
Syphilitic paralysis and muscular atrophy, diplopia and ptosis with, 107
Syphilitic lichen, 237
Syphilitic ghost, 567
Syrup of acacia, 697
Tannin, local use of, 495
Tannate of iron in diphtheria acid, 693
Tape-worm, carbolic acid for, 53
Tape-worm, ether for, 316
Tar, in bronchial catarrh and winter cough, value of, 625
Taxis, for strangulated hernia, new mode of, 154

Teeth, deafness cured by extraction of, 177
Teeth, simple method of removing in children, 729
Tendons, the suture of, 102
Tendons, ingrafting of, 434
Tepid baths in diseases of the chest, especially pulmonary phthisis, 152
Tetanus, by A. R. Kilpatrick, M. D., 18
Tetanus, two cases of idiopathia, 304
Tetanus, chloral and morphine in, 373
Tetanus, absolute repose in, 499
Tetanus twenty-eight days after injury, treated by chloral, 622
Thanks, our, 319
Therapeutical applications of eucalyptus globulus, 103
Therapeutic notes, 621
Theology, women in medicine, law and, 700
Thoracentesis, indications for, 419
Throat, necessity for active treatment in all acute diseases of the, 612
Throat, gargle for sore, 695
Throat, removal of foreign bodies from, by injections, 489
Tincture mur. ferri in nasal polypi, 295
Tincture iodine for cloasma uterina, 355
Tincture iron in erysipelas, external use of, 425
Tippler, the champion chloroform, 111
Tobacco, anti-emetic properties of, 416,
Tobacco, eucalyptus and, 493
Toe-nail, ingrowing, 692
Tonsilitis, external use of turpentine in, 51, 316
Tonsils, enlargement of, a cause of nightmare, 57
Tonic prescriptions, 114, 250
Tooth-ache drops, 180, 181, 182
Too good to be true, 311
Tooth-ache, bicarbonate of soda in, 429
Torticolis, treatment of, 430
Traumatic erysipelas, injections of carbolic acid in, 112
Traumatic inflammation of the liver, 693
Tremens delirium, treatment of, 171, 435
Trichaniæ in wild boars, 313
Tuberculosis not incurable, 104
Tumor of fœtus, perineal, an impediment to delivery, 615
Turpentine in tonsilitis, external use of, 51, 316
Turpentine an antidote to phosphorus, 171
Turpentine linament, transparent, 307
Twins, resemblance of, 55
Typhoid fever, diarrhœa of, 31, 356
Typhoid fever, 43, 169
Typhoid or enteric fever, causation of, 49
Typhoid fever, delirium , 49, 498
Typhoid fever, ulceration of vagina larynx, &c., in, 51
Typhoid fever, treatment of, 111
Typhoid fever, acidulated gargles in, 181, 488
Typhoid fever, treated by air baths, 251
Typhoid fever, new method of treating, 293
Typhoid fever, and broncho pneumonia, 340
Typhoid fever, digitalis in, 435
Typhoid fever in schools, cause and prevention of, 687
Typhoid fever, inunction in, 737
Ulcers, new method for healing, 37, 561
Ulcers, chronic, 244
Ulcers, a diagnostic hint in, 249
Ulcers, salicylic acid for, 309
Ulcers, sulphuret of carbon in chronic atonic, 438
Ulcers, dressing for, 621, 738
Ulceration, liquor ferri-chloride in uterine, 745
Ulceration of the vagina larynx and other mucous surfaces in typhoid fever, 51
Unchangeable-solution of quinia for hypodermic injection, 315
University publishing company, New York and Baltimore, 61
Unstopping a deaf ear, 118
Urine, incontinence of, in paralysis, 38
Urine, aspirator in retention of, 372
Urine, puncture of bladder in retention of, 428
Urine, retention and suppression of, by R. J. Preston, M. D., 459
Urine, incontinence of, 554, 623, 626
Urine, incontinence of, subcutaneous injections of strychnine in, 692
Urine, hematoxylen for alx. saline, 744
Uric acid, generators of in the organism, 663
Urticaria, mustard foot-bath in, 370, 750
Urticotus lichen, local treatment of, 567
Uteri, mamal dilatation of the os, as a means of inducing premature labor, by A. D. Sinclair, M. D., 207
Uterine diseases, hot water injections in, 26
Uterine neuralgia, 47
Uterine diseases, the rectum in its relation to, by Arthur W. Edis, M. D., 163
Uterine fibroid, successful Cæsarean section in a case of, 369
Uterus, polypus of the, treated by internal administration of ergot, by Daniel F. Collins, M. D., 286

Uterus, ergotin in fibroids of, 444
Uterus, displacements of the, 540
Uterus, case of retroversion of the, in sixth month of pregnancy, etc., by Patrick Booth, M. D., 594
Uterus, diminution of. after delivery, 686
Uterina, tincture of iodine for cloasma, 355
Vaccination, beneficial effect of, 39
Vaccination, puslute, diphtheria of a, followed by general diphtheria, 561
Vaccine, impure, 749
Vagina, ulceration of, in typhoid fever, 51
Vagina and uterus, case of double, by A. E. Hoadley, M. D., 661
Vagina pruritus, 234
Vaginal fistula cured by caustics, vesico, 481
Vaginal fistula, a new instrument for operating on vesico, 558
Valerianate of zinc in dysmenorrhœa, 751
Varix, treatment of, 745
Varices, by W. H. Taland, M. D., 274
Varicose veins treated by a new operation, 303
Varicose veins, treatment of, 575, 627
Varicocele, operation for, by the subcutaneous wire-loop, 545
Vehicle of disease, milk as a, 310
Veins, varicose, treated by a new operation, 303
Veins, varicose, treatment of, 575, 627,
Venesection, 401
Venous circulation? What are the causes of, by J. G. Knox, M. D., 582
Vesico vaginal fistula cured by caustics, 481
Vesico vaginal fistula, a new instrument for operating on, 558
Volitentes musca, by John Caston, M. D., 656
Vomiting from cough, to check, 56
Vomiting, enemata of bromide of potassium in obstinate, 492
Vomiting in pregnancy treated by dilatation of the os uteri, obstinate, 441, 628
Warts, 178, 498, 752
Warts, cure for, 372
Warm and hot water in surgery, by C. Henry Leonard, M. D., 607
Warren prize, the, 699
Water, lead in soda, 114
Water, warm and hot in surgery, by C. Henry Leonard, M. D., 607
Wet sheet in scarlatina, the, 227
Wet strapping, 613
Whooping cough, 242, 309, 745
Wine in fever? Why do we give, 44
Wind instruments, influence of use of, in chest affections, 565
Winter cough, value of tar in bronchial catarrh and, 625
Wire-loop, operation for varicocele by the subcutaneous, 545
Women, on the use of the binder in parturient, by P. H. Clarke, M. D., 513
Women in medicine, law and theology, 700
Wood, new process for rendering, incombustible, 255
Wood sorrel in epithelioma, 439
Worms, paralysis caused by, 100
Worms, 303
Wounds dressed with silicated cotton, 308
Wound, threatened symptoms from a punctured, by F. K. Bailey, M. D., 534
Wound of the stomach and kidney, bullet, 503
Xonthium strumarium, 500
Zinc, oxide of, in infantile diarrhœa, 620
Zinc, oxide of in diarrhœa, 627
Zaster, soothing application in herpes, 227

### COLLABORATORS AND CONTRIBUTORS.

GEORGIA.

James A. Long, M.D.
W. L. M. Harris, M.D.
H. L Battle, M.D.
W. C. Musgrove. M D
L B. Bouchelle, M.D.
Thomas Burdell, M.D.
E. H. W. Hunter, M.D.
V. S Hitt, M.D.
J. E. Pope, M.D.
J. C. Wisdom, M.D.
W. W. Leake, M.D.
S. G. White, M.D.
W. H. Hall, M.D.
G. D Case, M.D.
W. T. Grant, M.D.
J. M. Tules, M.D.
J. N. Cheney, M.D.
T. M. Shaw, M.D.
T. S. Mitchell, M.D.
H. B. Lee, M.D.
C. B. Nottingham, M.D.
W. L. Kirkpatrick, M.D.

KENTUCKY.

W. S. Taylor, M.D.
B. W. Stone, M.D.
Charles Kearns, M.D.

TEXAS.

S. M. Welch, M.D.
T. J. Heard, M.D.
L. R. Lincecum, M.D.

TENNESSEE.

J. D. Tucker, M.D.
F. A. Ramsey, M.D.
J. W. Hayne, M.D.
F. K. Bailey, M.D.

WEST VIRGINIA.

N. D. Tobey, M.D.

ALABAMA.

J. C. Knox, M.D.
Geo. F. Taylor, M.D.
J. B. Barnett, M.D.
S. M. Hogan, M.D.
J. T. Searcy, M.D.
A. C. Edwards, M D.
M. W. Morton, M.D.
J. D. Rush, M.D.
J. J. Grace, M.D.

NORTH CAROLINA.

J. B. Hughes, M.D.

S. S. Satchwell, M.D.

A. B. Pierce, M.D.

H. Kelly, M.D.

Geo. A. Foote, M.D.

E. A. Anderson, M.D

H. T. Bahnson, M.D.

John W. Booth, M.D.

R. S. Hallsey, M.D.

SOUTH CAROLINA.

E. T. McSwain, M.D.
G. M. Rivers, M.D.

OHIO.

J. Q. A. Hudson, M.D
C. H. Tidd, M.D.
J. C. Bishop, M.D.
W. P. Wells, M.D.

NEW YORK.

Ely Van DeWarker, M. D.
C. C. F. Gay, M.D.
J. S. Bailey, M.D.

COLORADO.

Wm. R. Whitehead, M.D.

VIRGINIA.

F. Horner, Jr., M.D.
R. J. Preston, M.D.
J. S. Wharton, M.D.
Sam'l P. Ripley, M.D.
E. A. Drewry, M.D.
Rob B. Tunstall, M.D.
W. F. Barr, M.D.
L. B. Anderson, M.D.
J. N. Upshur, M.D.
B. P. Reese, M.D.

MISSISSIPPI.

C. B. Gallaway, M.D.
Edward Lea, M.D.
S. V. D. Hill, M.D.
E. T. Henry, M.D.
T. P. Lockwood, M.D.
Robert Kells, M.D.
W. L. Lipscomb, M.D.
W. T. Beall, M.D.
A. G. Smythe, M.D.
J. M. Lewis, M.D.
J. H. Payne, M.D.
T. H. Mayo, M.D.
E. Cutter, M.D.

ARKANSAS.

A. D. Hodge, M.D.
J. K. Hodge, M.D.
A. W. Hobson, M.D.
C. D. Hodge, M.D.

INDIANA.

G. A. Dyer, M.D.

A. Patton, M.D.

Robert Robson, M D.

ILLINOIS.

John H. Weir, M.D.

OREGON,

W. M. Cusick, M.D.

